# Mortality inequalities measured by socioeconomic indicators in Brazil: a scoping review

**DOI:** 10.11606/s1518-8787.2022056004178

**Published:** 2022-09-30

**Authors:** Maria Yury Ichihara, Andrêa J.F. Ferreira, Camila S. S. Teixeira, Flávia Jôse O. Alves, Aline Santos Rocha, Victor Hugo Dias Diógenes, Dandara Oliveira Ramos, Elzo Pereira Pinto, Renzo Flores-Ortiz, Leila Rameh, Lilia Carolina C. da Costa, Marcos Roberto Gonzaga, Everton E. C. Lima, Ruth Dundas, Alastair Leyland, Maurício L. Barreto

**Affiliations:** I Fundação Oswaldo Cruz Centro de Integração de Dados e Conhecimentos para Saúde Salvador BA Brasil Fundação Oswaldo Cruz. Centro de Integração de Dados e Conhecimentos para Saúde. Salvador, BA, Brasil; II Universidade Federal da Bahia Instituto de Saúde Coletiva Salvador BA Brasil Universidade Federal da Bahia. Instituto de Saúde Coletiva. Salvador, BA, Brasil; III Universidade Federal da Bahia Escola de Nutrição Salvador BA Brasil Universidade Federal da Bahia. Escola de Nutrição. Salvador, BA, Brasil; IV Universidade Federal do Rio Grande do Norte Programa de Pós-Graduação em Demografia Natal RN Brasil Universidade Federal do Rio Grande do Norte. Programa de Pós-Graduação em Demografia. Natal, RN, Brasil; V Universidade Federal da Paraíba Departamento de Finanças e Contabilidade João Pessoa PB Brasil Universidade Federal da Paraíba. Departamento de Finanças e Contabilidade. João Pessoa, PB, Brasil; VI Universidade Federal da Bahia Instituto de Matemática e Estatística Salvador BA Brasil Universidade Federal da Bahia. Instituto de Matemática e Estatística. Salvador, BA, Brasil; VII Universidade Estadual de Campinas Departamento de Demografia Campinas SP Brasil Universidade Estadual de Campinas, Departamento de Demografia. Campinas, SP, Brasil; VIII Medical Research Council University of Glasgow Glasgow Scotland Medical Research Council. University of Glasgow, Glasgow, Scotland

**Keywords:** Mortality, trends, Geographic Locations, epidemiology, Socioeconomic Factors, Health Status Disparities, Review

## Abstract

**OBJECTIVE:**

Summarize the literature on the relationship between composite socioeconomic indicators and mortality in different geographical areas of Brazil.

**METHODS:**

This scoping review included articles published between January 1, 2000, and August 31, 2020, retrieved by means of a bibliographic search carried out in the Medline, Scopus, Web of Science, and Lilacs databases. Studies reporting on the association between composite socioeconomic indicators and all-cause, or specific cause of death in any age group in different geographical areas were selected. The review summarized the measures constructed, their associations with the outcomes, and potential study limitations.

**RESULTS:**

Of the 77 full texts that met the inclusion criteria, the study reviewed 24. The area level of composite socioeconomic indicators analyzed comprised municipalities (n = 6), districts (n = 5), census tracts (n = 4), state (n = 2), country (n = 2), and other areas (n = 5). Six studies used composite socioeconomic indicators such as the Human Development Index, Gross Domestic Product, and the Gini Index; the remaining 18 papers created their own socioeconomic measures based on sociodemographic and health indicators. Socioeconomic status was inversely associated with higher rates of all-cause mortality, external cause mortality, suicide, homicide, fetal and infant mortality, respiratory and circulatory diseases, stroke, infectious and parasitic diseases, malnutrition, gastroenteritis, and oropharyngeal cancer. Higher mortality rates due to colorectal cancer, leukemia, a general group of neoplasms, traffic accident, and suicide, in turn, were observed in less deprived areas and/or those with more significant socioeconomic development. Underreporting of death and differences in mortality coverage in Brazilian areas were cited as the main limitation.

**CONCLUSIONS:**

Studies analyzed mortality inequalities in different geographical areas by means of composite socioeconomic indicators, showing that the association directions vary according to the mortality outcome. But studies on all-cause mortality and at the census tract level remain scarce. The results may guide the development of new composite socioeconomic indicators for use in mortality inequality analysis.

## INTRODUCTION

Observed within different sociodemographic groups^1^, the inverse relationship between low socioeconomic status and mortality is a well-established fact in the literature and has mostly been analyzed by single-variable socioeconomic indicators such as income, education, wealth, race/ethnicity, marital status, social class, and occupation^4,5^. Composite socioeconomic indicators such as the Human Development Index, deprivation scores, and social-vulnerability indexes^6^ have also been used to study mortality inequalities in populations. These more complex measures broaden the knowledge on socioeconomic disparities, especially in analyses that consider different geographical levels, such as municipalities, or other small areas.

In Brazil, several studies provide evidence of higher mortality rates in more impoverished areas^9^. Many are the composite socioeconomic measures available at the municipal level, such as the Social Vulnerability Index (*Índice de Vulnerabilidade Social* – IVS)^12^, and the Municipal Human Development Index (MHDI)^13^ – still, mortality rates can be highly heterogeneous^14^, making more disaggregated analyses desirable. Smaller spatial units like districts or census tracts (which include districts), however, often lack socioeconomic information^15^, resulting in few studies on mortality inequality at this level of analysis.

Using indicators to analyze mortality inequalities at different geographical levels has been most beneficial for researchers and health policy makers to identify the risks of death in population groups and to define public policies and interventions^16,17^. Mapping the construction of composite socioeconomic indicators, and their association with mortality outcomes at different geographical levels in Brazil, is of paramount importance to guide the development of new composite indicators and their use in studies analyzing mortality inequalities. As such, this study summarizes the literature on the relationship between composite socioeconomic indicators and mortality in different geographical areas of Brazil.

### Specific Research Questions

To do so, we formulated the following research questions:

**Research question 1:** Which composite socioeconomic indicators are most used to understand mortality inequalities across different Brazilian geographical areas?

**Research question 2:** What are the characteristics of these composite measures of area-level socioeconomic indicators, and are there any limitations to understanding geographical mortality inequalities in Brazil?

## METHODS

This scoping review follows the Meta-analysis of Observational Studies in Epidemiology (MOOSE) guidelines and is reported according to the Preferred Reporting Items for Systematic Reviews and Meta-Analyses Statement extension for Scoping Reviews (PRISMA-ScR)^18^. Its protocol was submitted and published on the Open Science Framework (OSF) (https://osf.io/vmt9f/).

We used the population, concept, and context framework to define our research question^19^. Population was defined as individuals who had died in Brazil, considering all-cause, or specific causes of death in any age range; concept was understood as the aggregate measures of socioeconomic position; and the context was the geographical level in Brazil (i.e., state, municipality, census tract level, districts, and others)^18^.

### Eligibility Criteria

This scoping review included papers that: i) were published in peer-reviewed journals between January 1, 2000 and August 31, 2020; ii) had cross-sectional, cohort, case-control, and ecological study designs; iii) analyzed the relationship (i.e., association or descriptive relationship) between socioeconomic status and all-cause, cause-specific, or prevalence mortality rates; iv) outcomes for the general population could be provided by administrative or primary data, without age group or geographical area level restriction. Articles that exclusively accounted for single measures of the socioeconomic condition, reviews, trials, intervention studies, editorials, comments, and case reports were excluded.

### Outcomes

Primary outcomes consisted of all-cause mortality, while secondary outcomes comprised cause-specific mortality – both defined according to the International Classification of Diseases (ICD). Data were processed from baseline to follow-up. If a study reported multiple follow-ups, only the most recent data was included.

### Information Sources and Search Strategy

We performed a bibliographic search on August 31, 2020, in the Medline, Scopus, Web of Science, and Latin American and Caribbean Health Science Literature (Lilacs) databases. The authors, aided by an experienced librarian, drafted the search strategy bellow, used for PubMed/Medline:

(Poverty [MESH] OR deprivation [MESH] OR “socioeconomic position” [TIAB]) AND (Mortality [MESH] OR death* OR lethality OR fatality) AND Brazil.

An adapted version of this search strategy was drafted and used for the Web of Science, Scopus, and Lilacs databases. Final search results were exported into EndNote, and two blinded authors removed any duplicates. All references were managed in EndNote X7. We did not search for gray literature.

### Selection of Evidence Sources and Data Charting

Three pairs of reviewers independently evaluated the titles, abstracts, and full texts of the selected articles. Prior to standardized data extraction, the reviewers were trained on key study descriptors to harmonize the extraction: i) article identification (language, authors, year, and journal of publication); ii) composite socioeconomic measure (name, data source, variables used, level of analysis, and geographical coverage); iii) mortality outcomes (cause of death, age group, data sources, and type of measure); iv) statistical analysis; and v) main findings. Disagreements between reviewers were resolved by means of discussion, and in collaboration with a third reviewer as necessary. We did not estimate the agreement rate for the reviews. Finally, two pairs of reviewers verified all the previously extracted information. Information was summarized in tables and boxes.

### Summary of Results

Data analysis was carried out following the narrative summary approach^20^. Results were tabulated considering the publication year, geographical coverage, and mortality outcomes of the study. The summary included: the different socioeconomic indicators available, the all-cause and specific cause of death, and the main findings and limitations –as well as critical points the authors failed to address – of the selected studies. Information was summarized according to the population coverage of the socioeconomic measure, area level, composition and scale of the socioeconomic inequality measures incorporated, data and information sources used, and analytic methods used to describe the relationship between socioeconomic inequalities and mortality outcomes.

## RESULTS

We retrieved a total of 806 papers – of which we removed 208 duplicates and excluded other 521 following title screening, leaving a total of 77 full-text articles for assessment. Figure describes the exclusion process during the full-text review. Most studies were excluded for not including a composite socioeconomic measure (n = 33), or not being peer-reviewed articles (n = 12). After screening, 24 articles remained for the scoping review.

**Figure:**
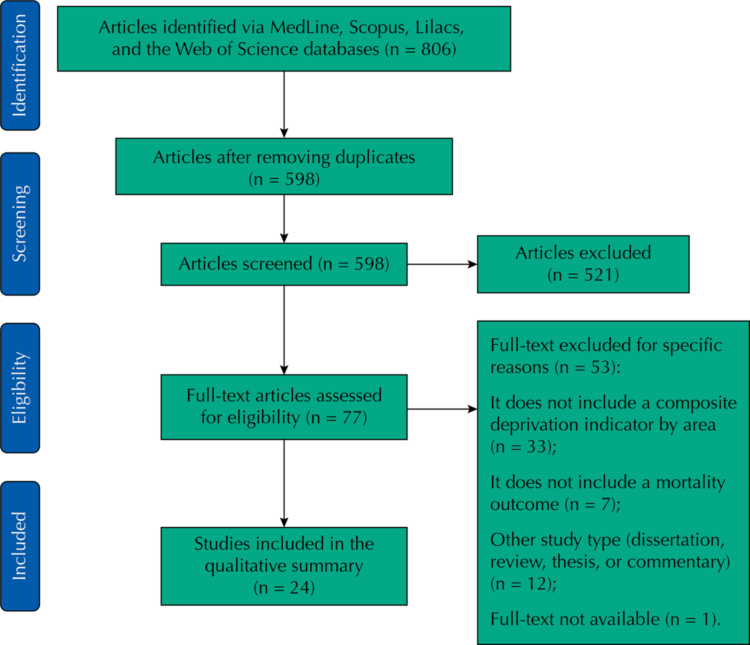
Flow diagram for the scoping review process.

Evenly distributed across two study periods, most studies covered the municipal level (n = 16) (Table). The articles reviewed measure socioeconomic indicators at the country (n = 2), state (n = 2), municipal (n = 6), district (n = 5), and census tract (n = 4) level, as well as other geographic areas (n = 5). Mortality outcomes mainly comprised cause-specific rates (n = 19), and age-specific mortality outcomes (n = 11) (Table).

**Table:** Characterization of the studies included in the scoping review.

Characterization	n	%
Year of publication		
2000–2010	12	50.0
2011–2020	12	50.0
Geographical coverage of the study		
Country	4	16.7
State	4	16.7
Municipality	16	66.6
Area level of socioeconomic inequity indicators		
Country	2	8.3
State	2	8.3
Municipality	6	25.0
Districts	5	20.8
Census tracts	4	16.6
Others	5	20.8
Mortality outcomes^a^		
All-cause mortality	2	8.3
Cause-specific (may be broken down, according to results)	19	79.2
Age-specific mortality outcomes	11	45.8

^a^ Non-mutually exclusive categories.

Box 1 presents the selected articles organized according to composite socioeconomic measures and mortality outcomes. Some studies assessed mortality outcomes at the small area level (census tract)^21^, making it difficult to generalize their results for the whole of Brazil, since the combined composite socioeconomic measure was only constructed for a given geographical area (Box 1).

**Box 1:** Summary of the selected studies according to socioeconomic inequities and mortality and main findings.

Author(s)/year	Composite indicator	Variables/domains	Source/year of variables/domains	Mortality measure	Main findings
Country					
Machado et al.^37^ (2019)	Human Development Index (HDI)	Income, schooling, and longevity	Not specified	Suicide, homicide, and road traffic accidents mortality rates	HDI mortality rates were most evident in the poorest quintiles.
Alarcão et al.^39^ (2020)	Human Development Index and Social Vulnerability Index	Income, schooling, and longevity	Not specified	Age-specific suicide mortality rate (15–19, 20–24, and 25–29 years old)	Socioeconomic deprivation was an important determinant of suicide in younger people and significantly influenced high-risk groups for suicide mortality rates.
State					
Guimarães et al.^40^ (2013)	Socioeconomic status	Gross Domestic Product *per capita*; average household income *per capita*; Gini Index; adults with family income < 1/2 minimum wage	Not specified	Age-adjusted mortality rate (≥ 20 years old) for colorectal cancer	Mortality rates according to gender were directly related to lower socioeconomic status.
Ribeiro et al.^27^ (2007)	Social Exclusion Index (SEI)	Poverty, employment, income, literacy, years of schooling, population aged ≤ 19 years, and violence	Brazilian Demographic Census (2000)	Age-adjusted leukemia mortality rate (birth to 4, 5–9, 10–14, and 15–19 years old)	Social inequality was negative correlated with leukemia mortality rates in both genders; higher significant decreases in the more developed states.
Municipal					
Schuck-Paim et al.^38^ (2019)	Human Development Index	Income, schooling, and longevity	Not specified	Pneumonia mortality rate (≤ 59 months)	Pneumonia mortality rate was declined modestly and statistical significantly in municipalities with a high percentage of extreme childhood poverty, and a higher proportion of low maternal schooling.
Drachler et al.^34^ (2014)	Social Vulnerability Index (IVS-5)	Household conditions: income, water distribution, garbage collection, bathroom, illiteracy in people aged >15, and overcrowding	Brazilian Demographic Census (2010)	Child mortality rate	The most vulnerable municipalities had higher hospitalization rates for sensitive. Primary care conditions, and a higher infant mortality rate than the least vulnerable municipalities.
Medeiros et al.^28^ (2012)	Socioeconomic Development Index (*Índice de Desenvolvimento Socioeconômico* - IDESE)	Four thematic blocks: schooling; income; sanitation and household conditions; and health	Brazilian Demographic Census (2001)	CVD mortality rate (ischemic, hypertensive, and cerebrovascular)	Direct relationship between average mortality rate and IDESE in municipalities with 5,000–15,000 inhabitants. IDESE variables only partially explained the differences in CVD mortality rates in socioeconomically similar municipalities.
Faria and Santana^35^ (2016)	Material Deprivation Index (*Índice de Privação Material* - IPM)	Illiteracy among women of reproductive age, households without indoor sanitary facilities, and unemployment	Brazilian Demographic Census (2010)	Infant mortality rate (< 1 year)	High infant mortality rates in municipalities with high material deprivation.
Alves et al.^41^ (2020)	Social Determination Indicator	Dimension 1: vulnerable to poverty and schooling; Dimension 2: household income	Municipal Department of Health Surveillance	Tuberculosis mortality risk	A worse social condition, such as low schooling levels and poverty, increased tuberculosis mortality risk by three times.
Bonfim et al.^36^ (2020)	Social Deprivation Index (SDI)	Household conditions, no nominal monthly income, and illiterate head of household	Brazilian Demographic Census (2010)	Fetal and infant mortality rate	High fetal and infant mortality rates found in areas with poor living conditions, where SDI showed spatial dependence (I = 0.18; p = 0.014) of clusters.
Districts					
Silva et al.^29^ (2008)	Composite Social Deprivation Indicator (*Indicador Composto de Carência Social* – ICS)	Household conditions, schooling, and income	Brazilian Demographic Census (2000)	All-cause, cause-specific, and age-adjusted (> 60 years old) mortality rate	Positive correlation between social deprivation and cause-specific deaths. Districts with extreme social deprivation had a 2.9 times higher risk of death due to traffic accidents, and 3.9 times higher risk of pneumonia in older adults.
Araújo et al.^45^ (2005)	Socioeconomic condition	Healthcare unit, urban infrastructure and services, safety, schooling, household building pattern, and afforestation	Municipal Planning Secretariat (1999)	External cause mortality rate	Spatial distribution of external cause mortality rates showed differences in socioeconomic levels. Risk of death from homicides and traffic accidents was higher in the low and medium-low socioeconomic strata. Areas in the middle and high socioeconomic strata presented higher mortality rates from traffic accidents.
Bassanesi et al.^30^ (2008)	Socioeconomic condition	Education, population income, density, external cause mortality rate, aging and fertility rates, and infant mortality	Brazilian Demographic Census (2000)	Average CVD mortality rate (ischemic and cerebrovascular)	Early CVD mortality rate was 2.6 times higher in districts classified as the worst social stratum. Among districts in the most extreme deprivation strata, RR reached 3.3 for CVD, and 3.9 for cerebrovascular diseases. 62% of early deaths were in the worst stratum.
Campos et al.^42^ (2000)	Socioeconomic composition of districts	Houses with a single-family; households with infrastructural conditions; head of household monthly income, and favela census tracts related to the total number of sectors in each neighborhood	Department of Health Information censuses and maps - Oswaldo Cruz Foundation (1995)	Infant mortality rate (neonatal and post-neonatal) and proportional mortality by cause groups	Infant mortality rate showed a dispersed spatial distribution, without direct relation to the socioeconomic profile. The flow of children between their residences and place of death shows a movement that starts in the most impoverished areas towards the wealthier ones, which have a greater number of health facilities.
Oliveira et al.^31^ (2010)	Composite Deprivation Index	Head of the family’s income and schooling, and household conditions	Brazilian Demographic Census (2000)	Standardized cause-specific mortality rate (circulatory system, neoplasms, respiratory system, deaths from external causes, perinatal infections, and infectious and parasitic diseases)	Most cases of aggression (86%) occurred in the most deprived groups. There was no statistically significant correlation between socioeconomic levels and perinatal, cancer, respiratory, or parasitic mortality.
Census tract					
Peres et al.^21^ (2011)	Social Exclusion/Inclusion Index (*Índice de Exclusão/Inclusão Social *– IEI)	1. Autonomy: income, employment, and destitution; 2. Quality of life: access to basic services, housing infrastructure and travel; 3. Human development: schooling, longevity, and risk of death; 4. Equity: income and literacy of women heads of households	Brazilian Demographic Census (2000) and other national and municipal sources: SEADE (2000), PRO-AIM (2000), Fipe (2000), Embraesp (2000), and Metro (1997)	All-cause homicide mortality rate and by type of weapon, gender, race/skin color, age, and areas of social exclusion/inclusion	The gradient of homicide mortality rates increased as the degree of social exclusion increased. There was a very sharp decline in homicide mortality rates in extreme and high exclusion (-79.3% and -71.7%, respectively). There was also a decline in the mean and degree of social exclusion (-59.1% and -61.9%, respectively).
Vilela et al.^22^ (2008)	Social Deprivation Indicator (*Indicador de Carência Social* – ICS)	Household conditions and schooling/family head’s income	Brazilian Demographic Census (2000)	Infant mortality rate from infectious and parasitic diseases as the underlying and/or associated cause of death	There was a 48% higher risk of children under one year dying from infectious and parasitic diseases in the stratum of highest social deprivation.
Antunes et al.^23^ (2008)	Socioeconomic status	Unemployment, insufficient schooling, family head’s academic qualifications, and Human Development Index	Brazilian Demographic Census (2000)	Oral and pharyngeal cancer mortality rate stratified by gender, age, year, underlying cause, and inner-city area of residence	The distribution of rate terciles at the area level highlighted a spatial gradient of mortality in the city. In the poorer areas (second and third terciles), higher mortality rates prevailed.
Silveira and Junger^24^ (2018)	Social Development Index	Household conditions, illiteracy among people aged 10 to 14, and family head’’ income	Brazilian Demographic Census (2010)	Ischemic heart disease and cerebrovascular disease mortality rate	Greener sectors (third quartile) had 6.7% (95%CI: 3.5–9.8) and 4.7% (95%CI: 1.2–8.0) less mortality due to ischemic heart disease and cerebrovascular disease, respectively. Protective effect of green space was more significant for lower socioeconomic status (8.6%; 95%CI: 1.8–15.0). For cerebrovascular disease mortality rate, a protective effect was observed at the lowest socioeconomic levels (9.6%; 95%CI: 2.3–16.5).
Others					
Bastos et al.^32^ (2009)	Urban Quality Index (*Índice de Qualidade Urbana* - IQU)	Schooling, income, housing infrastructure, and urban services infrastructure	Brazilian Demographic Census (2000)	External cause mortality rate (traffic accident, homicide, and suicide)	Homicide victims were young, black, male, residing in the poorest urban areas, and had lower IQU values. Suicides and traffic accidents affected older adults, white women, and residents of the wealthiest areas, with the highest IQU scores.
Belon and Barros^33^ (2011)	Global Socioeconomic Level Score	Family head’s income and schooling	Brazilian Demographic Census (2000)	Life expectancy	Life expectancy for men and women was 6.9 and 5.5 years lower in impoverished areas than in areas of higher socioeconomic status. Social inequalities in life expectancy at birth decreased between 2000– 2005 as groups of lower socioeconomic status gained more years of life.
Teixeira et al.^25^ (2002)	Index of Living Conditions (*Índice de Condições de Vida* - ICV)	Income, schooling, overcrowding, sanitation, and subnormal clusters	Brazilian Demographic Census (1991)	Mortality due to infectious and parasitic diseases: proportional mortality, mortality rate, and the standardized and specific mortality ratio	The highest mortality rates due to infectious and parasitic diseases occurred in poorer living conditions.
Macedo et al.^26^ (2001)	Living Condition Status	Economic capital (income in minimum salaries); cultural capital (head of family’s schooling level)	Brazilian Demographic Census (1991)	Homicide mortality rate	The highest homicide mortality rate was found in the city’s most impoverished areas. Estimated homicide related to the risk of death was 2.9 (1991) in the worst living conditions, and 5.1 (1994) for better conditions.
Lotufo and Benseñor^44^ (2009)	Social Exclusion Index	Concentration of young people, literacy, years of schooling, formal employment, violence, and inequality	Not specified	Stroke mortality by gender	Odds of death by stroke was 2.0 times higher in districts with higher social exclusion. A similar pattern was found for ischemic and hemorrhagic strokes in both genders. It had a negative correlation between income and proportional stroke mortality.

CVD: cardiovascular disease; Seade: *Sistema Estadual de Análise de Dados* (State Data Analysis System Foundation); PRO-AIM: *Programa de Aprimoramento das Informações de Mortalidade* (Mortality Information Improvement Program); Fipe: *Fundação Instituto de Pesquisas Econômicas *(Foundation Institute of Economic Research); Embraesp: *Empresa Brasileira de Estudos de Patrimônio* (Brazilian Company of Heritage Studies); Metro: The São Paulo Metropolitan Company.

Most articles (n = 16) elaborated composite socioeconomic indicators using Brazilian Demographic Census data: two, ten, and four articles, respectively, were written using data from the 1991^25,26^, 2000^21^, and 2010^24,34^ censuses. Other data sources included the Municipal Planning Secretariat, the State System of Statistical Data Analysis Foundation (*Fundação Sistema Estadual de Análise de Dados Estatísticos* – SEADE), the Mortality Information Improvement Program of São Paulo (*Programa de Aprimoramento das Informações de Mortalidade* – PRO-AIM), the Institute for Economic Research Foundation (*Fundação Instituto de Pesquisas Econômicas* – Fipe), the Brazilian Agency for Heritage Studies, and the Metropolitan Company of São Paulo (*Companhia Metropolitana de São Paulo* – Metrô).

Four studies used global indicators as measures, such as the Human Development Index (HDI), which considers variables related to income, education, and longevity^23,37^; and the Gross Domestic Product (GDP), i.e., the sum of all final goods and services produced in a given period of time^28^. One study^40^ used the Gini Index, a single global index that evaluates income inequality, in conjunction with other variables to compose socioeconomic inequality measures (Box 1).

The papers reviewed used a wide variety of variables to create composite socioeconomic measures, most commonly *per capita* or household income (n = 17)^22^, schooling (n = 17)^21^, household status (n = 12)^22,24,25,28,29,32,34^, and employment (n = 5)^21,23,27,35,44^ (Box 1). Social classes^42^, inequality^44^, proportion of extreme poverty^41^, and quality of life^21^ were less frequently used (Box 2). We also described the grouping of variables in their respective dimensions for construction of the combined measures in each article, as well as the source and year of the data used to measure socioeconomic inequalities (Box 1).

**Box 2:** Summary of limitations reported by the selected studies.

Author(s)/ Year	Limitations
Peres et al.^21^ (2011)	Lack of temporal data on the potential social determinants of homicide decline made it impossible to infer its causes. There were no discussions on the limitations resulting from the social exclusion/inclusion index.
Vilela et al. (2008)^22^	The limitations considered were intra-aggregate heterogeneity, inter-group mobility, and the underreporting of infant deaths.
Antunes et al. (2008)^23^	Different ways of measuring variables in statistical censuses in Barcelona and São Paulo. As an ecological study, it does not consider the relevant variation in individual socioeconomic characteristics. Another limitation is the relatively simple analytical scheme, which disregards non-linear relationships between mortality and socioeconomic status.
Silveira and Junger^24^ (2018)	Use of secondary data is a limiting factor in this study, as is the possibility of ecological studies.
Machado et al.^37^ (2019)	The short period analyzed.
Schuck-Paim et al.^38^ (2019)	Despite the synthetic control method used to detect the benefits of pneumococcal conjugate vaccine introduction and explicitly designed to minimize confounding, the ecological study design may have disregarded other uncontrolled factors that can affect the estimates.
Medeiros et al^ 28^ (2012)	Use of secondary data is a limiting factor in this study. Information may not be completely reliable and represents population averages as it is an ecological study.
Ribeiro et al.^27^ (2007)	Ecological study that had to consider “ecological fallacy.” No individual assessment of socioeconomic status was performed in the study, and the smallest unit analyzed (state) was too large to represent a neighborhood effect.
Drachler et al.^34^ (2014)	Does not mention limitations.
Silva et al.^29^ (2008)	The associations found may be stronger due to spatial aggregation (neighborhood). The districts of Recife still have significant social heterogeneity, with wealth areas existing alongside pockets of poverty. Moreover, this study characterized mortality using an indicator created by other authors. Research shows that synthetic indicators do not capture the different nuances of social reality.
Bassanesi et al.^30^ (2008)	Does not mention limitations.
Campos et al.^42^ (2000)	Does not mention limitations.
Oliveira et al.^43^ (2010)	Considering the nature of the aggregate measure, a neighborhood classified with the highest deprivation does not always have the worst rates on all variables analyzed. The high variation in population composition (between 2,500 and 60,000 people) across districts was not considered. Regarding statistical analysis, no mortality smoothing techniques was performed, as it was not possible to assess the effect of deprivation on mortality. Use of the 2000 census to obtain socioeconomic indicators may result in limitations in understanding previous years.
Belon and Barros^33^ (2011)	As a unit of analysis and area of residence, a limitation of this study is that its results do not necessarily reflect the situation of those belonging to each socioeconomic stratum.
Teixeira et al.^25^ (2002)	Does not mention limitations.
Macedo et al.^26^ (2001)	The stratification adopted in the study, although performed by aggregating similar zones, has several limitations due to the particular heterogeneity of the urban area of Salvador. Problems related to the quality of information were also studied.
Bastos et al.^32^ (2009)	An important limitation of ecological studies is that the relationship between two variables does not necessarily reflect the situation under study. Administrative regions may have caused degrees of heterogeneity due to the specific characteristics of each neighborhood.
Faria and Santana (2016)^35^	Use of secondary data can be considered a limiting factor in this study.
Lotufo and Benseñor^44^ (2009)	Does not mention limitations.
Araújo et al.^45^ (2005)	Lack of data on living conditions disaggregated by neighborhood prevented the generation of a weighted indicator for classification according to more specific sociodemographic variables. Moreover, the quality of violent death records restricted a more comprehensive understanding.
Alves et al.^41^ (2020)	Limiting factors comprise the use of secondary data and the fact that deaths due to more severe forms of the outcome were not verified.
Guimarães et al.^40^ (2013)	An ecological design that needed to measure the variables as proxies: income does not directly interfere with colorectal cancer. It can promote conditions to decrease exposure to risk factors, such as diet (primary prevention), and establish early diagnosis (secondary prevention).
Alarcão et al.^39^ (2020)	Use of secondary data and collinear variables (schooling, income, and employment), which may impair the strength of the association, are limitations in this study.
Bonfim et al.^36^ (2020)	Given the difference in coverage of the Mortality Information System throughout Brazil, the use of secondary data is a possible limitation in this study.

Most articles (n = 17) used the Mortality Information System (*Sistema de Informação em Mortalidade* – SIM), from the Unified Health System IT Department (*Departamento de Informática do Sistema Único de Saúde* – Datasus) as the source of mortality data^23,25,27^. Other studies used mortality systems from the Municipal Health Department^21,22,26,32,33,42,43^, and records from the Forensic Medicine Institute (*Instituto Médico Legal* – IML)^45^. Only one study did not specify the source of mortality data used^30^. The source of population count data used as the denominator for the mortality rates was either the Brazilian Institute of Geography and Statistics (*Instituto Brasileiro de Geografia e Estatística* – IBGE) censuses or the Live Birth Information System (*Sistema de Informação sobre Nascidos Vivos* – SINASC) (Box 1).

Mortality rates were mostly presented in non-standardized form^24,30,32,37,45^, and commonly calculated for a specific age group, such as infant mortality^34^ and mortality of older adults^29^ (Box 1). One study evaluated life expectancy^33^. To tackle the different frequency distributions in diverse populations, some authors chose age-standardized rates^25,27,40,43,46^, or stratification of rates by different age groups and other population characteristics, such as sex and race/ethnicity^21,23,30,38,39,44^. The studies either estimated the rates for each year or measured the average mortality rate between periods. They also used proportional mortality linked to causes or age groups^25,42^. Cause-specific mortality rates comprised external causes^22,25,38,41^, infectious and parasitic diseases^22,25,38,41^, and chronic and degenerative diseases^23^ (Box 1). Age-specific mortality rates consisted mainly of children^34^. Only two studies estimated all-cause mortality rates^29,33^ (Box 1).

Most studies (n = 14) presented descriptive statistics and/or spatial distribution analysis, correlating mortality outcomes with the classification of socioeconomic inequalities by area. Areas with the worst socioeconomic characteristics had higher mortality indicators for the following causes: all-cause mortality^43,45^, external causes^43^, suicide^37,39^, homicide^21,26,37,43,45^, fetal mortality^36^, infant mortality^34^, respiratory system diseases^29^, circulatory system diseases^43^, stroke^44^, infectious and parasitic diseases^22,25,29^, diarrhea^29^, malnutrition^29^, gastroenteritis^29^, and oropharyngeal cancer^23^.

Higher mortality rates due to colorectal cancer^40^, leukemia^27^, a general group of neoplasms^29^, traffic accidents^32,45^, and suicide^32^, in turn, were observed in less deprived areas and/or those with more significant socioeconomic development. Medeiros et al.^28^ (2012) showed that the variables in the socioeconomic development measure only partially explained the differences in mortality rates due to cardiovascular diseases in a group of socioeconomically similar municipalities, being more strongly associated with other determinants.

Studies found higher mortality risks for tuberculosis (RR = 2.9)^41^, pneumonia (RR = 3.9)^29^, cardiovascular diseases (RR = 3.3)^30^, cerebrovascular diseases (RR = 3.9)^30^, stroke (OR = 2.0)^45^, homicide (RR = 5.1)^26^, traffic accidents (RR = 2.9)^29^, and infectious and parasitic diseases among children (RR = 1.48)^22^ in more deprived areas compared with less deprived areas. But no statistically significant association was found between mortality rates and indicator measures, such as the Composite Social Deprivation Indicator (*Indicador Composto de Carência Social* – ICS)^29^, the socioeconomic composition of districts^43^ and the Composite Deprivation Index^43^ (Box 2). Moreover, a study evaluating life expectancy at birth showed that this variable was 6.9 and 5.5 years less, respectively, for men and women living in impoverished areas, compared with those living in less deprived areas^33^.

### Limitations Discussed by the Studies

Studies based on the ecological approach^22^ reported some disadvantages regarding the assessment of mortality inequalities using composite socioeconomic measures (Box 2). As these studies were not designed to find an association between socioeconomic factors and mortality at the individual level^32,47,48^, and the potential explanation pointed to a decrease in heterogeneous spatial contexts, particularly in large areas and populations^22,26,32,33,43^, their results may not necessarily reflect the situation of individuals belonging to each socioeconomic strata (Box 2). Other limitations concerned the use of secondary data, even if from official governmental sources, which could mask underreporting of death, and the difference in SIM coverage between the different areas studied^28,35^. As for analytical and measurement strategies, the studies discussed limitations in the availability of mortality data in censuses^36,37,41^, the difficulty of using rate smoothing methods in smaller areas^44^ and more robust methods to assess the association between mortality and the composite socioeconomic measures used^23,39^ (Box 2).

### Study Limitations Noted by this Review

Some of the studies reviewed did not discuss possible study limitations, as described above^21,25,30,34,42,44^. Other important limitations also went unaddressed, such as the presence of a garbage code – i.e., causes of deaths that should not be considered underlying causes of deaths^49^ –, and ill-defined causes of death (IDCD), which could influence the results when correction and distribution, respectively, are not performed^49^. We must also point out the lack of discussion regarding the uncertainty of mortality data in some Brazilian regions (north and northeast) and at small area levels, such as the census tract. The quality of the mortality information system also varies across these regions and could be a source of bias and therefore should be discussed. Since the composite socioeconomic measures used in mortality iniquity studies also vary, these should be addressed as the differences in definitions and concepts (i.e., deprivation, vulnerability, socioeconomic status, and poverty) could influence the interpretation of their findings.

## DISCUSSION

To our knowledge, this is the first study to provide a comprehensive overview of the available literature on composite socioeconomic measures and mortality in different geographical areas of Brazil, and to identify the methodological challenges in analyzing these associations. Our main findings reveal that while some of the composite socioeconomic measures used in mortality studies covered the entire country, they were limited by the area of analysis – the municipality. Only four studies used the census tract as the small area level to assess mortality data, but their results were restricted to specific municipalities^21^. Cause-specific mortality outcomes (i.e., external causes, chronic and degenerative diseases, infectious and parasitic diseases) were the most frequent.

Most studies used descriptive and spatial analysis to estimate the relationship between socioeconomic measures and mortality outcomes, with a few articles employing regression analysis to estimate this association. None of the studies reviewed used a gradient analysis to estimate the aforementioned relationship. Some articles presented a gradient analysis according to socioeconomic status, where the lowest socioeconomic status had the highest mortality rates and the greatest increase in some mortality outcomes, or specific causes of death, as observed in other countries^6,8^. But we also found studies citing lower mortality rates in the lower socioeconomic strata, particularly for cancers^21,27,37,38^.

Currently in Brazil, we have a variety of socioeconomic indices that are construed based on different socioeconomic and geographical variables, and with different concepts. Thus, none of the development or vulnerability indicators, or similar concepts are available nationally for the entire country at different geographic levels^15^. Besides, current measures address concepts other than socioeconomic conditions. Although deprivation, poverty, and vulnerability broadly refer to a person’s impoverished state compared to society as a whole, they are theoretically distinct. Vulnerability refers to the risk of experiencing a decline in well-being, or in the quality of living conditions. Similarly, material deprivation can be defined as lack of income, and other resources^50^. Poverty, in turn, is measured by alternative concepts based on subsistence, basic needs, and relative deprivation^51^.

Socioeconomic measures are popular and widely used in studies focused on assessing health outcomes and economic and social development results^1^. In Brazil, however, we have a lack of studies using standardized measures covering the entire country, as well as those related to all-cause mortality – since most of the studies reviewed here used cause-specific mortality. Since the distribution of all-cause and cause-specific mortality rates is a key metric for assessing population health, a better understanding of the impact of lower socioeconomic conditions on different levels and mortality trends can help policymakers plan and develop priorities for allocating health resources^52^.

In Brazil, the register of deaths is compulsory and such records are reported in national information systems, such as the Ministry of Health’s Mortality Information System (SIM) and the Civil Registry Statistics System (RC). Moreover, the last IBGE Demographic Census, carried out in 2010, gathered information on deaths for the entire population of Brazil included in it^53^. Deaths in Brazil require certification by a physician, and are defined according to ICD codes^54^.

Despite great advances in recent decades in the quality of mortality information systems in Brazil, we still have significant underreporting of deaths, especially in less-developed regions of the north and northeast, added to the differences by sex, age, and area of residence^55,56^. In small areas, the issue of significant data uncertainty regarding the number of deaths makes mortality estimates even more innacurate^14^. Consequently, studies that use mortality indicators without correcting for underreporting may not effectively measure mortality rates in the region and instead report false and misleading associations. Similarly, the last decade saw a reduction in the percentage of garbage codes in the mortality information system, which demonstrates its improved quality^57^. Also, after inclusion of the IDCD reclassification results in the country’s official statistics published in 2010, the percentage of IDCD decreased from 8.6% to 7.0% among reported deaths. Such percentage, however, is still relatively high, presenting significant disparities between states and regions. This variation also occurs intra-regionally, with IDCD percentages close to 30.0% in some states’ macro-regions^54^. In 2015, for example, studies observed an estimated 97.2% of deaths recorded in the mortality system^31^. Despite improvements in the quality and integrity of the SIM database over time, we still find heterogeneity in the frequency and completeness of reports^57,58^. Moreover, underestimation and mis-coding of deaths is more problematic in older adults and young children groups^31,59^.

All-cause and cause-specific mortality analyses should therefore be carried out using methods that consider correction for deaths by the remaining IDCD. Since the magnitude of these causes can be affected, this can introduce biases in comparisons between locations with different IDCD percentages, and between different socioeconomic groups. Due to issues with information quality, analyses of trends and leading causes of mortality in many low- and middle-income countries, such as Brazil, are usually restricted to areas with a higher socioeconomic status or larger cities; while places with the poorest quality of information on deaths have the heaviest disease burden. Such an issue requires further exploration in new studies to better understand the relationship between inequalities and mortality rates across the country^54^.

Death distribution reflects the countries’ socioeconomic development contexts^60,61^. Historical data from developed countries show that as their socioeconomic and health conditions improved, mortality rates tended to consistently decrease^60,61^ – trend that has yet to become homogeneous for middle- and low-income countries, which possess substantial regional differences^60,61^. People of low socioeconomic status, defined by their *per capita* and/or household income, schooling, employment status, type of household, and internal and associated conditions, etc., are more likely to die younger than those of high socioeconomic status^62^.

Low socioeconomic status is consistently associated with an increased risk of premature and all-cause mortality. The reviewed studies show that the worst all-cause and cause-specific mortality outcomes were associated with the worst socioeconomic measures. The mechanisms by which this social status can negatively affect health are diverse and include difficulty purchasing food, inadequate housing/neighborhoods, and barriers to accessing health and social services. Other social determinants may also explain these findings, such as: alcohol and tobacco consumption; different cultural standards related to healthy and unhealthy behavior; stress and low self-esteem associated with low socioeconomic status, which can lead to harmful physiological changes; less social capital in impoverished communities; and environmental factors (i.e., high crime/violence rates, lack of public transportation, polluted roads, fast food outlets, and waste disposal sites^4,63,64^). Regarding difficulties in accessing health services, studies report issues with prenatal care and early childhood care services, control of infectious diseases, and lack of access to dental services. They also point to the existence of social selection, a form of reverse causality in which disease causes, or deepens, social inequalities^65^.

Despite consistent reference to low socioeconomic status as a predictor for mortality, the aggregate scale of socioeconomic inequity on mortality in small areas in Brazil is still unclear. Existing socioeconomic measures only estimate social and economic inequalities down to the municipal level for the entire country^12,13^. And those few measures available for disaggregated levels (i.e., census sector) are usually restricted to a single municipality or state. When evaluating a municipality, a better general socioeconomic condition may thus mask smaller pockets of extreme poverty. At the census tract level, the socioeconomic deprivation measure can identify areas with higher and lower mortality risks within the same municipality. Ultimately, identifying small areas with the worst mortality outcomes can guide the reallocation of resources and implementation of public policies.

### Strengths and Limitations

To our knowledge, the present study is the first to review the literature on the relationship between composite socioeconomic indicators and mortality outcomes at different geographic levels in Brazil, and to identify the methodological challenges in analyzing these associations. Since we used a standard data extraction form for each paper included in the scoping review, our data should be as robust and standardized as possible. As the evidence reviewed may have been limited by the variety of terms/concepts related to composite socioeconomic measures such as deprivation, vulnerability, poverty, and socioeconomic status, our study also has limitations. Nevertheless, we consider that our search strategy, and the databases searched, included the main scientific literature on this topic. Our scoping review did not require a full risk of bias as it was not designed to produce an estimate of the effect of inequality on mortality. Instead, we summarized the limitations discussed by each study, highlighting any possible limitation that could influence the findings and was not reported.

## CONCLUSIONS

This scoping review showed that studies have found higher rates, or higher percentages of increased mortality rates, in areas considered to be more impoverished, vulnerable, or have less socioeconomic development – despite remaining methodological omissions in measuring mortality disparities at lower geographic levels. Area-based deprivation indicators can facilitate linking information for socioeconomic and health conditions in the same area. The possibility of using a concise deprivation measure available for the lowest geographic level (census tract) across the country is essential for assessing health outcomes and for implementing public policies to reduce mortality inequalities in Brazil. Area-based deprivation indicators can also contribute to monitoring progress against the Sustainable Development Goal targets for different health outcomes.

## References

[1] Rocha SMR (2007). Pobreza no Brasil: afinal, de que se trata?.

[2] Gonçalves SL (2015). Vulnerabilidade das famílias à pobreza: uma análise empírica para seis regiões metropolitanas: 2002 to 2011.

[3] Ribas-Fitó N, Sala M, Kogevinas M, Sunyer J (2001). Polychlorinated biphenyls (PCBs) and neurological development in children: a systematic review. J Epidemiol Community Health.

[4] Bosworth B (2018). Increasing disparities in mortality by socioeconomic status. Annu Rev Public Health.

[5] Williams J, Allen L, Wickramasinghe K, Mikkelsen B, Roberts N, Townsend N (2018). A systematic review of associations between non-communicable diseases and socioeconomic status within low- and lower-middle-income countries. J Gob Health.

[6] Ruiz JI, Nuhu K, McDaniel JT, Popoff F, Izcovich A, Criniti JM (2015). Inequality as a powerful predictor of infant and maternal mortality around the world. PloS One.

[7] Sánchez-Garrido N, Aguilar-Navarro SG, Ávila-Funes JA, Theou O, Andrew M, Pérez-Zepeda MU (2021). The Social Vulnerability Index, mortality and disability in Mexican middle-aged and older adults. Geriatrics.

[8] McCartney G, Popham F, Katikireddi SV, Walsh D, Schofield L (2017). How do trends in mortality inequalities by deprivation and education in Scotland and England & Wales compare? A repeat cross-sectional study. BMJ Open.

[9] Anele CR, Hirakata VN, Goldani MZ, Silva CH (2021). The influence of the municipal human development index and maternal education on infant mortality: an investigation in a retrospective cohort study in the extreme south of Brazil. BMC Public Health.

[10] Jaen-Varas D, Mari JJ, Asevedo E, Borschmann R, Diniz E, Ziebold C (2019). The association between adolescent suicide rates and socioeconomic indicators in Brazil: a 10-year retrospective ecological study. Braz J Psychiatry.

[11] Ribeiro AG, Downward GS, Freitas CU, Chiaravalloti F, Cardoso MRA, Latorre MRDO (2019). Incidence and mortality for respiratory cancer and traffic-related air pollution in São Paulo, Brazil. Environ Res.

[12] Instituto de Pesquisa Econômica Aplicada (2021). Atlas da vulnerabilidade social.

[13] Programme United Nations Development (2021). Atlas of human development in Brazil.

[14] Schmertmann CP, Gonzaga MR (2018). Bayesian estimation of age-specific mortality and life expectancy for small areas with defective vital records. Demography.

[15] Ichihara MYT, Ramos D, Rebouças P, Oliveira FJ, Ferreira AJF, Teixeira C (2018). Area deprivation measures used in Brazil: a scoping review. Rev Saude Publica.

[16] Exeter DJ, Zhao J, Crengle S, Lee A, Browne M (2017). The New Zealand Indices of Multiple Deprivation (IMD): a new suite of indicators for social and health research in Aotearoa, New Zealand. PloS One.

[17] Leyland AH, Dundas R, McLoone P, Boddy FA (2007). Cause-specific inequalities in mortality in Scotland: two decades of change. A population-based study. BMC Public Health.

[18] Tricco AC, Lillie E, Zarin W, O’Brien KK, Colquho H, Levac D, Moher D (2018). PRISMA Extension for Scoping Reviews (PRISMA-ScR): checklist and explanation. Ann Intern Med.

[19] Aromataris E, Munn Z (2020). JBI manual for evidence synthesis.

[20] Popay J, Roberts H, Sowden A, Petticrew M, Arai L, Rodgers M (2006). Guidance on the conduct of narrative synthesis in systematic reviews: A product from the ESRC Methods Programme. Version 1.

[21] Peres MFT, Vicentin D, Nery MB, Lima RS, Souza ER, Cerda M (2011). Queda dos homicídios em São Paulo, Brasil: uma análise descritiva. Rev Panam Salud Publica.

[22] Vilela MBR, Bonfim C, Medeiros Z (2008). Mortalidade infantil por doenças infecciosas e parasitárias: reflexo das desigualdades sociais em um município do Nordeste do Brasil. Rev Bras Saude Mater Infant.

[23] Antunes JLF, Borrell C, Pérez G, Boing AF, Wünsch-Filho V (2008). Inequalities in mortality of men by oral and pharyngeal cancer in Barcelona, Spain and São Paulo, Brazil, 1995-2003. Int J Equity Health.

[24] Silveira IH, Junger WL (2018). Green spaces and mortality due to cardiovascular diseases in the city of Rio de Janeiro. Rev Saude Publica.

[25] Teixeira MG, Meyer MA, Costa MCN, Paim JS, Silva LMV (2002). Mortalidade por doenças infecciosas e parasitárias em Salvador - Bahia: evolução e diferenciais intra-urbanos segundo condições de vida. Rev Soc Bras Med Trop.

[26] Macedo AC, Paim JS, Silva LMV, Costa MCN (2001). Violência e desigualdade social: mortalidade por homicídios e condições de vida em Salvador, Brasil. Rev Saude Publica.

[27] Ribeiro KB, Lopes LF, Camargo B (2007). Trends in childhood leukemia mortality in Brazil and correlation with social inequalities. Cancer.

[28] Medeiros CRG, Meneghel SN, Gerhardt TE (2012). Desigualdades na mortalidade por doenças cardiovasculares em pequenos municípios. Cienc Saude Colet.

[29] Silva VL, Leal MCC, Marino JG, Marques APO (2008). Associação entre carência social e causas de morte entre idosos residentes no Município de Recife, Pernambuco, Brasil. Cad Saude Publica.

[30] Bassanesi SL, Azambuja MI, Achutti A (2008). Premature mortality due to cardiovascular disease and social inequalities in Porto Alegre: from evidence to action. Arq Bras Cardiol.

[31] Oliveira ATR (2018). Sistemas de estatísticas vitais no Brasil: avanços, perspectivas e desafios.

[32] Bastos MJRP, JdA Pereira, Smarzaro DC (2009). Ecological analysis of accidents and lethal violence in Vitória, Southeastern Brazil. Rev Saude Publica.

[33] Belon AP, Barros MBA (2011). Reduction of social inequalities in life expectancy in a city of Southeastern Brazil. Int J Equity Health.

[34] Drachler ML, Lobato MAO, Lermen JI, Fagundes S, Ferla AA, Drachler CW (2014). Desenvolvimento e validação de um índice de vulnerabilidade social aplicado a políticas públicas do SUS. Cienc Saude Colet.

[35] Faria R, Santana P (2016). Variações espaciais e desigualdades regionais no indicador de mortalidade infantil do estado de Minas Gerais, Brasil. Saude Soc.

[36] Bonfim CV, Silva APSC, Oliveira CM, Vilela MBR, Freire NCF (2020). Spatial analysis of inequalities in fetal and infant mortality due to avoidable causes. Rev Bras Enferm.

[37] Machado DB, Pescarini JM, Araújo LFSC, Barreto ML (2019). Austerity policies in Brazil may affect violence related outcomes. Cienc Saude Colet.

[38] Schuck-Paim C, Taylor RJ, Alonso WJ, Weinberger DM, Simonsen L (2019). Effect of pneumococcal conjugate vaccine introduction on childhood pneumonia mortality in Brazil: a retrospective observational study. Lancet Glob Health.

[39] Alarcão ACJ, Dell’ Agnolo CM, Vissoci JR, Carvalho ECA, Staton CA, Andrade L (2020). Suicide mortality among youth in southern Brazil: a spatiotemporal evaluation of socioeconomic vulnerability. Braz J Psychiatry.

[40] Guimarães RM, Rocha PGM, Muzi CD, Ramos RS (2013). Increase income and mortality of colorrectal cancer in Brazil, 2001-2009. Arq Gastroenterol.

[41] Alves JD, Arroyo LH, Arcoverde MAM, Cartagena-Ramos D, Berra TZ, Alves LS (2020). Magnitud de los determinantes sociales en el riesgo de mortalidad por tuberculosis en el Centro-Oeste de Brasil. Gac Sanit.

[42] Campos TP, Carvalho MS, Barcellos CC (2000). Mortalidade infantil no Rio de Janeiro, Brasil: áreas de risco e trajetória dos pacientes até os serviços de saúde. Rev Panam Salud Publica.

[43] Oliveira DC, Barreira ÁS, Trunk MT, Guzmán AF (2010). Efecto de las desigualdades socioeconómicas en la mortalidad de la ciudad de Fortaleza, Ceará, Brasil durante el año 2007. Rev Esp Salud Publica.

[44] Lotufo PA, Benseñor IM (2009). Stroke mortality in Brazil: one example of delayed epidemiological cardiovascular transition. Int J Stroke.

[45] Araújo EM, Araújo TM, Santana F (2005). Distribuição desigual da mortalidade por causas externas: avaliação de aspectos socioeconômicos. Rev Baiana Saude Publica.

[46] Guimarães EA (2013). O processo de implementação do Programa Minha Casa Minha Vida para a população de baixa renda: o caso de Viçosa, MG.

[47] Silva RM, Sousa GS, Vieira LJES, Caldas JMP, Minayo MCS (2018). Suicidal ideation and attempts of older women in Northeastern Brazil. Rev Bras Enferm.

[48] Ribeiro F, Leist A (2020). Who is going to pay the price of Covid-19? Reflections about an unequal Brazil. Int J Equity Health.

[49] Ministério da Saude (BR), Secretaria de Vigilância em Saúde, Departamento de Análise da Situação de Saúde (2009). Manual para investigação do óbito com causa mal definida.

[50] Dutta I, Foster J, Mishra A (2011). On measuring vulnerability to poverty. Soc Choice Welf.

[51] Townsend P (1987). Deprivation. J Soc Policy.

[52] Benedetti MSG, Saraty SB, Martins AG, Miranda MJ, Abreu DMX (2019). Evaluation study of the garbage codes research project in the northern region of Brazil. Rev Bras Epidemiol.

[53] Lima EEC, Gonzaga MR, Freire FHMA, Queiroz BL (2021). Alternative information sources on deaths in Brazil in the context of the COVID-19 pandemic.

[54] França E, Teixeira R, Ishitani L, Duncan BB, Cortez-Escalante JJ, Morais OL (2014). Ill-defined causes of death in Brazil: a redistribution method based on the investigation of such causes. Rev Saude Publica.

[55] Queiroz BL, Lima EEC, Freire FHMA, Gonzaga MR (2020). Temporal and spatial trends of adult mortality in small areas of Brazil, 1980-2010. Genus.

[56] Queiroz BL, Gonzaga MR, Vasconcelos AMN, Lopes BT, Abreu DMX (2020). Comparative analysis of completeness of death registration, adult mortality and life expectancy at birth in Brazil at the subnational level. Popul Health Metrics.

[57] Teixeira RA, Naghavi M, Guimarães MDC, Ishitani LH, França EB (2019). Quality of cause-of-death data in Brazil: Garbage codes among registered deaths in 2000 and 2015. Rev Bras Epidemiol.

[58] Lima EEC, Queiroz BL (2014). Evolution of the death registry system in Brazil: associations with changes in the mortality profile, under-registration of death counts, and ill-defined causes of death. Cad Saude Publica.

[59] Szwarcwald CL, Leal MC, Esteves-Pereira AP, Almeida WS, Frias PG, Damacena GN (2019). Avaliação das informações do Sistema de Informações sobre Nascidos Vivos (SINASC), Brasil. Cad Saude Publica.

[60] Ezzati M, Pearson-Stuttard J, Bennett JE, Mathers CD (2018). Acting on non-communicable diseases in low-and middle-income tropical countries. Nature.

[61] Wang X, Auchincloss AH, Barber S, Mayne SL, Griswold ME, Sims M (2017). Neighborhood social environment as risk factors to health behavior among African Americans: The Jackson Heart Study. Health Place.

[62] Braveman P, Gottlieb L (2014). The social determinants of health: it’s time to consider the causes of the causes. Public Health Rep.

[63] Silva VL, Cesse EAP, Albuquerque MFPM (2014). Social determinants of death among the elderly: a systematic literature review. Rev Bras Epidemiol.

[64] Skalická V, Ringdal K, Witvliet MI (2015). Socioeconomic inequalities in mortality and repeated measurement of explanatory risk factors in a 25 year follow-up. PloS One.

[65] Acheson D (1998). Independent inquiry into inequalities in health report.

